# Inter- and Intra-Day Comparisons of Smartphone-Derived Heart Rate Variability across Resistance Training Overload and Taper Microcycles

**DOI:** 10.3390/ijerph18010177

**Published:** 2020-12-29

**Authors:** Tyler D. Williams, Michael R. Esco, Michael V. Fedewa, Phillip A. Bishop

**Affiliations:** 1 Department of Kinesiology, Samford University, Birmingham, AL 35229, USA; 2 Department of Kinesiology, University of Alabama, Tuscaloosa, AL 35487, USA; mresco@ua.edu (M.R.E.); mvfedewa@ua.edu (M.V.F.); pbishop@ua.edu (P.A.B.)

**Keywords:** athlete monitoring, powerlifting, bench press, strength

## Abstract

The purposes of this study were: (1) to determine if smartphone-derived heart rate variability (HRV) could detect changes in training load during an overload microcycle and taper, and (2) to determine the reliability of HRV measured in the morning and measured immediately prior to the testing session. Twelve powerlifters (male = 10, female = 2) completed a 3-week resistance training program consisting of an introduction microcycle, overload microcycle, and taper. Using a validated smartphone application, daily measures of resting, ultra-short natural logarithm of root mean square of successive differences were recorded in the morning (LnRMSSD_M_) and immediately before the test session (LnRMSSD_T_) following baseline, post-overload, and post-taper testing. LnRMSSD_M_ decreased from baseline (82.9 ± 13.0) to post-overload (75.0 ± 9.9, *p* = 0.019), while post-taper LnRMSSD_M_ (81.9 ± 7.1) was not different from post-overload (*p* = 0.056) or baseline (*p* = 0.998). No differences in LnRMSSD_T_ (*p* < 0.05) were observed between baseline (78.3 ± 9.0), post-overload (74.4 ± 10.2), and post-taper (78.3 ± 8.0). LnRMSSD_M_ and LnRMSSD_T_ were strongly correlated at baseline (ICC = 0.71, *p* < 0.001) and post-overload (ICC = 0.65, *p* = 0.010), whereas there was no relationship at post-taper (ICC = 0.44, *p* = 0.054). Bland–Altman analyses suggest extremely wide limits of agreement (Bias ± 1.96 SD) between LnRMSSD_M_ and LnRMSSD_T_ at baseline (4.7 ± 15.2), post-overload (0.5 ± 16.9), and post-taper (3.7 ± 15.3). Smartphone-derived HRV, recorded upon waking, was sensitive to resistance training loads across an overload and taper microcycles in competitive strength athletes, whereas the HRV was taken immediately prior to the testing session was not.

## 1. Introduction

Athlete monitoring is a strategy that many strength and conditioning coaches use to assess fatigue and adaptation with regard to training [1,2]. While intense training is a major physiological stressor experienced by athletes, other factors such as sleep, nutrition, and emotional state may add to the overall stress imposed. The accumulation of these stressors will require sufficient recovery, or else noticeable decreases in performance may be experienced [2]. An effective monitoring tool for athletes in training would need to be sensitive enough to detect important perturbations in homeostasis and provide adequate information needed to alter training loads to optimize recovery. Current methods of monitoring training loads can be classified by their ability to monitor internal or external load. External load represents the total work performed during training [1] and can be quantified using the training volume. Monitoring the external load has the potential to provide the feedback needed for making informed decisions; however, it lacks the ability to assess the physiological and psychological responses to training, which is referred to as internal load.

One physiological measure that is gaining popularity as a monitoring tool is heart rate variability (HRV). HRV is the variation in time intervals between consecutive heartbeats and provides a physiological marker of autonomic regulation [3]. The root mean square of successive R–R interval differences (RMSSD) is a parasympathetically-derived HRV marker that has been shown to be the most reliable metric of athletic monitoring. This non-invasive measure has been proposed to provide an objective assessment of training status [4,5,6] and training load [7,8,9]. Using HRV to monitor the recovery status or guide exercise prescription [10] has previously been limited to evaluation in laboratory settings because of the need for lengthy recording procedures and specialized equipment [11]. Recently, the development of portable smartphone applications capable of accurately measuring HRV in field settings has made this assessment more practical and cost-effective [12]. Previous research has demonstrated that smartphone-derived HRV is sensitive to detect changes in training loads in soccer players [7,8] and competitive swimmers [9]. For best results, it is recommended that athletes record HRV upon waking [4], though this approach may provide poor compliance. As such, it may be more practical to record HRV immediately prior to a training session. However, it is unknown if HRV measures taken prior to training will be as sensitive to a recovery marker compared to the recommended morning measures. 

Furthermore, while HRV has been shown to be an effective, non-invasive tool to monitor fatigue and recovery in endurance and sport team athletes [6,7], investigations in strength athletes are limited. Chen and colleagues examined HRV in competitive weightlifters 72 h following a 2 h resistance training session [13]. Frequency-domain measures of HRV reflected recovery status and weightlifting performance following the training bout. While this indicates that HRV monitoring may be effective for objectively assessing fatigue and adaptation in strength athletes, the recording methods utilized in their study would be impractical for field application. Smart-phone derived HRV measures provide a more efficient and practical approach to monitoring athlete preparedness during periods of intensive resistance training; however, more research is warranted to determine the efficacy of this method. Additionally, smartphone applications allow for HRV measures to be recorded immediately before training, which could lead to better athlete compliance. For these reasons, smartphone-derived HRV appears to be an appealing monitoring tool for guiding the training process. Currently, it remains unknown whether HRV reflects the training and recovery state following a resistance training microcycle and taper. Furthermore, whether HRV measured prior to a training bout is as sensitive for monitoring recovery as the preferred method of measuring upon waking [4] remains to be elucidated. Therefore, the purposes of this study were two-fold: (1) to determine the effect of an overload microcycle and taper on smartphone-derived HRV; and (2) to examine the reliability between HRV measures taken upon waking and those taken just prior to training. We hypothesized that smartphone-derived HRV would decrease following the overload microcycle and return to baseline following the taper. Furthermore, we hypothesized that there would be a difference between morning HRV and pre-testing HRV, and that morning HRV would be a better indicator of daily preparedness.

## 2. Materials and Methods 

### 2.1. Study Design

This study examined the effect of an intensified week of resistance training and taper on HRV and investigated the reliability between two HRV measures, immediately upon waking and immediately prior to a testing session. During the initial visit to the Exercise Physiology Lab, participants were familiarized with the HRV recording procedures. Additionally, written instructions were provided to ensure consistent measurement procedures. Participants completed a 3-week resistance training program consisting of introduction, overload, and taper microcycles. HRV measures were recorded at two time points on the 5th day of each microcycle. The first measurement was self-recorded by each participant upon waking and the second measurement was self-recorded with the help of a trained research assistant upon arriving at the training facility. In addition to measuring HRV, the participants completed a bench press one-repetition maximum (1RM) and bench press repetition-to-failure (RTF) assessment in order to determine the changes in sport-specific performance across overload and taper microcycles. 

### 2.2. Participants

Fifteen powerlifters (men: n = 12; women: n = 3) were recruited to participate in this study; however, 3 participants (men: n = 2; women: n = 1) were excluded from the analysis due to noncompliance with the HRV measurement protocol. An a priori power analysis (G power; ANOVA: repeated measures) revealed that a minimum of 6 participants were necessary to detect a 3.9 unit (lnRMSSDx20) difference in HRV (effect size = 0.35, α = 0.05, power = 80%, correlation among variables = 0.85, non-centrality parameter = 14.7, critical F = 4.4). The effect size and correlation used in the analysis were extracted from Flatt et al. [8]. Twelve participants completed the study; 10 men (height: 179.4 ± 4.2 cm; body mass: 96.9 ± 11.3; body fat %: 23.3 ± 5.3; relative bench press 1RM: 1.5 ± 0.2) and 2 women (height: 164.1 ± 4.7 cm; body mass: 71.7 ± 12.6; body fat %: 33.8 ± 1.0; relative bench press 1RM: 0.8 ± 0.0). The participants were competitive powerlifters and recreational powerlifters. Competitive powerlifters were defined as individuals who have previously competed in a sanctioned powerlifting competition (n = 8), whilst recreational powerlifters (n = 4) were defined as those who had not competed, but performed each of the powerlifts (back squat, bench press, and deadlift) at least once a week with the intent of increasing exercise-specific maximal strength. Participants were recruited from the university and a local powerlifting training center. All participants were healthy, non-smoking volunteers who met the criteria for exercise participation according to American College of Sports Medicine guidelines [14]. To minimize any confounding effects associated with age-related differences in skeletal muscle recovery between younger and older individuals, participants were all between the ages of 18–40 years.

All prospective participants completed an exercise screening questionnaire, PAR-Q, and health history questionnaire to determine if they met the inclusion criteria and were healthy for study participation [14]. To qualify for inclusion in the study, the participants had to have at least one year of resistance training experience and meet the national qualifying requirements established by the United States Powerlifting Association [15]. The powerlifting totals comprised of the participant’s highest squat, bench press, and deadlift were gathered from a previous competition or test session. All experimental procedures were conducted in accordance with the Declaration of Helsinki and approved by the University Institutional Review Board (16-017-ME). 

### 2.3. Procedures

During the initial visit, participants were informed of study procedures and provided written consent. After consent was obtained, the participants’ standing height was measured to the nearest 0.1 cm using a stadiometer (SECA 67310, SECA^®^, Chino, CA, USA) and the weight was measured to the nearest 0.1 kg using a digital scale (Tanita BWB-800, Tanita^®^, Arlington Heights, IL, USA). Body composition was assessed using dual-energy X-ray absorptiometry (Lunar Prodigy, General Electric Healthcare, Madison, WI, USA). Participants were provided with instruction regarding the resistance training program and performance measures administered during the study. Furthermore, participants were instructed on how to rate their perceived recovery and perceived exertion during the study. 

Within one week after the initial visit, the participants reported to the weight training facility to begin the 3-week resistance training program. The introduction microcycle consisted of 3 non-consecutive days over a 5-day period using a total-body program (Table 1). Training loads were determined using the rating of perceived exertion (RPE) scale based on repetitions in reserve [16]. This scale has been validated as a method of gauging resistance training intensity in novice and experienced lifters [16]. All exercise sets during the introduction microcycle were performed between an RPE of 6 to 8. On the fifth day of the introduction week (BL), baseline performance assessments were performed before completing the third resistance training session.

Two days after baseline testing, the participants began an overload week of resistance training, with the purpose of overreaching. Resistance training was performed on 4 consecutive days using a total-body program (Table 1). Daily undulations in training volume and intensity were used to incorporate the high-load and high-volume training as a means to induce central and peripheral fatigue. All sets were performed using repetition maximums and training loads were adjusted to ensure all sets were performed to muscular exhaustion. On the fifth day of the overload week (PostOL), participants completed the same assessments administered during the initial baseline testing session. To minimize any confounding effects for the time of day, the participants completed the post-overload test session during the same time of day as the initial baseline testing. Following the post-overload test session, the participants received 48 h of rest before tapering. 

The taper week consisted of two non-consecutive days of training over a 4-day period. Each day contained the same total body exercises used in the overload week; however, the total training volume was substantially reduced (Table 1). In an attempt to enhance performance, training intensity remained high during the taper [17,18]. Training loads were adjusted to 90% and 85% of the average load used during days 3 and 4, respectively, of the overload week. Post-taper performance measures were recorded on the fifth day of the taper week (PostTP). Each participant performed the same measures at the same time of day as performed during the BL and PostOL.

Prior to arrival at each performance testing session, the participants were instructed to refrain from caffeine for at least 12 h, and alcohol at least 24 h prior to testing. Before participating in the performance testing, the participants rated their perceptual recovery using the Perceived Recovery Status (PRS) scale. After indicating their perceived recovery, participants completed a full-body, dynamic warmup that was standardized for each session. All training sessions were monitored by a certified strength and conditioning specialist.

### 2.4. Heart Rate Variability (HRV)

Participants received the equipment needed to measure daily HRV during their initial visit. HRV was self-recorded using a smartphone application [12] with a pulse-wave finger sensor (PWFS) (ithlete^TM^, HRV Fit Ltd., Southampton, UK) that inserted into the headphone outlet of a smartphone or tablet device [19]. The smartphone application processed the R–R intervals and calculated the log-transformed root mean square of successive R–R intervals (LnRMSSD). For easier interpretation, the application multiplied the LnRMSSD by twenty to convert it to a value on a ~100-unit scale [12]. The application did not allow for the manual inspection of the R–R intervals, however, it was equipped with an irregular beat detection and a correction process [20].

Morning HRV (LnRMSSD_M_) was recorded upon waking and elimination. The PWFS was connected to the smartphone device and the participants’ left index finger was inserted into the PWFS. Following a brief stabilization period, a 55 s recording of HRV was taken in a seated position with the participant’s left hand within 20 cm of their chest. Participants were allowed to breathe at their own pace during the measurement period, since LnRMSSD has been shown to be consistent under paced or spontaneous breathing [21]. Pre-testing HRV (LnRMSSD_T_) was recorded using the same procedures as soon as participants arrived at the testing facility.

### 2.5. Internal and External Loads

The PRS scale ranges from 0 to 10 with 0–2 representing poor recovery and anticipating poor performance, 4–6 consisting of moderate recovery and expecting normal performance, and 7–10 representing high recovery and expecting increased performance [22]. The participants were informed of the purpose of the PRS scale and read specific instructions on how to interpret the scale. The PRS scale was used each day during each training and testing session to assess perceptual recovery. Training loads were determined by calculating the weekly volume load (sets × repetitions × load) for all resistance exercises performed. In addition to the volume load, training loads were quantified by collecting a session rating of perceived exertion (sRPE), a metric that is capable of quantifying a global rating of internal load [2]. Each participant reported their sRPE 15 min following the completion of the training session.

### 2.6. Assessment of Bench Press Performance

After the completion of a dynamic warmup, participants completed a 1RM bench press assessment. All bench press assessments were performed on an instrumented bench press (Forza Super Bench, Forza Strength Systems, Spokane Valley, WA, USA) using a 20.4 kg powerlifting competition barbell (Rogue Fitness, Columbus, OH, USA). Participants were instructed on how to perform the bench press to meet United States Powerlifting Association standards [15]. Hand position on the barbell was recorded for each participant and was maintained consistent for each trial. Participants began the assessment by performing five progressive warm-up sets in the bench press before attempting a 1RM. The first set consisted of 5 repetitions with an unloaded barbell, followed by 3 repetitions at 40%, 2 repetitions at 55%, and 1 repetition each at 70% and 85% of previously acquired 1RM. During each bench press attempt, the participants un-racked the barbell with assistance from a spotter. After receiving a secure hand off, the barbell was lowered to the chest and held motionless with a definite and visible pause. Once the primary investigator determined that the bar was motionless, a verbal “press” command was given and the participant proceeded to press the barbell to the lockout position. Participants were given verbal encouragement to press the barbell with maximal velocity on each repetition. The mean concentric velocity (MCV) was measured and recorded for each of the warm up repetitions using a linear position transducer (GymAware PowerTool, Kinematic Performance Technology, Canberra, Australia), which has been previously validated for measuring barbell velocity [23]. The MCV for the load–velocity profile was determined by averaging the mean velocities during the warm-up sets at 40%, 55%, 70%, and 85% 1RM and was used for analysis.

Two minutes after completing the one repetition at 85% of 1RM, the participants began 1RM bench press attempts. The weight for each attempt was selected by the primary investigator using measured repetition velocity and perceptual feedback (i.e., RPE) from the participant. The velocity feedback was used to objectively assess each attempt and subsequent loads were selected based on the reported ACV for competitive powerlifters in the bench press [24]. Additionally, a 1RM was determined based on the methods of Zourdos and colleagues [16]: (1) the participant reported a 10 RPE and the primary investigator agreed a subsequent attempt would not be successful with a 2.3 kg load increase or (2) the participant reported a 9 or 9.5 RPE followed by a failed attempt with a 2.3 kg load increase. If the participant failed to complete the concentric portion of the lift or did not pause on the chest, the attempt was deemed unsuccessful. 

Ten minutes after a successful 1RM bench press, the participants completed a bench press RTF assessment with 70% of 1RM. Participants were instructed to adhere to the same bench press guidelines as performed during the 1RM attempts. Spotters were available to provide verbal encouragement and safely rack the bar as the participant reached muscular failure. Muscular failure was determined by the inability to complete the concentric phase of the bench press.

### 2.7. Data Analysis

Data were analyzed using SPSS Statistics version 23.0 (IBM Corporation, Armonk, NY, USA) and Jamovi (version 0.9, Sydney, Australia), and Excel Office 365 (Microsoft Corporation, Redmond, WA, USA). The Shapiro–Wilk test was used to assess data normality. A 2 × 3 repeated measures analysis of variance (ANOVA) was used to compare the mean differences between LnRMSSD_M_ and LnRMSSD_T_ across BL, PostOL, and PostTP. When appropriate, a Tukey post hoc analysis was used to determine where the mean differences occurred. A one-way repeated measures ANOVA was used to compare bench press 1RM, MCV, and RTF across BL, PostOL, and PostTP, and a Tukey post hoc analysis was used to determine where differences occurred. For the perceived measures (sRPE and PRS) and non-normally distributed data, a Friedman’s test was used to compare differences and pairwise comparisons with *p*-value adjustments were used for post hoc analyses. Cohen’s *d* effect sizes [25] were calculated and interpreted using the following thresholds: 0 to 0.2 (trivial), 0.2 to 0.6 (small), 0.6 to 1.2 (moderate), 1.2 to 2.0 (large), >2.0 (very large) [26]. 

Changes (Δ, post-test–pre-test) in outcome variables across microcycles were quantified and Pearson’s correlations were used to determine the relationship between ΔLnRMSSD and Δbench press performance. Intra-day reliability between LnRMSSD_M_ and LnRMSSD_T_ were analyzed using an intraclass correlation coefficient (ICC). The ICC and Pearson correlations were interpreted using the following thresholds: ≤0.30 (small), 0.31 to 0.49 (moderate), 0.50 to 0.69 (large), 0.70 to 0.89 (very large), and ≥0.90 (near perfect) [26]. Additionally, Bland–Altman plots were generated to assess the agreement between LNRMSSD_M_ and LNRMSSD_T_ at each of the time points [27]. Data are presented as mean ± standard deviation unless otherwise indicated, and the level of significance was set at *p* ≤ 0.05.

## 3. Results

### 3.1. Internal and External Load Comparisons

External loads and internal loads during the introduction, overload, and taper microcycles are displayed in Table 2. There was a significant main effect for the total training volume (*p* < 0.001). The training volume during the overload was higher than the introduction microcycle (*p* < 0.001, *d* = 3.41) and taper microcycle (*p* < 0.001, *d* = 3.85). During the taper, the total volume load was lower than the introduction (*p* < 0.001, *d* = −0.64). There was a significant main effect for the session perceived exertion (*p* < 0.001). Overload sRPE values were higher than introduction values (*d* = 3.36) and taper microcycle (*p* < 0.001, *d* = 4.17). During the taper, the sRPE values were lower compared to the introduction (*p* < 0.001, *d* = −1.24). There was a significant main effect for perceived recovery (*p* < 0.001). PRS scores at PostOL were lower than BL (*p* < 0.001, *d* = −1.76) and PostTP (*p* < 0.001, *d* = −2.37). At PostTP, PRS was higher compared to BL (*p* < 0.001, *d* = 1.21). 

### 3.2. Inter-Day LnRMSSD Comparisons

**Figure 1:**
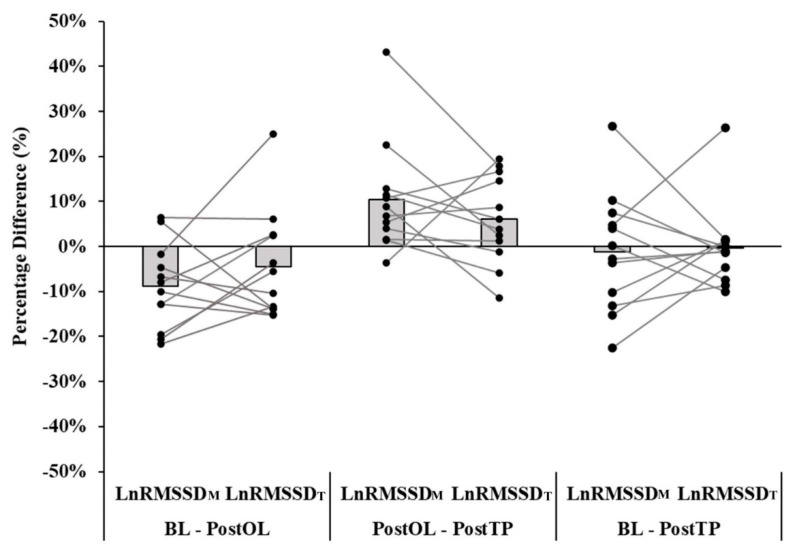
Individual changes in the natural logarithm of root mean square of successive R–R interval differences during the morning (LnRMSSD_M_) and pre-testing (LnRMSSD_T_) time points between baseline (BL), post-overload (PostOL), and post-taper (PostTP) test days.

Means and SDs for LnRMSSD_M_ and LnRMSSD_T_ across each time point are presented in Table 2. There was no condition × time interaction (*p* = 0.363; n^2^*_p_*_=_ 0.088) and no main effect for condition (*p* = 0.090, n^2^*_p_*_=_ 0.239). There was a significant main effect for time (*p* = 0.007; n^2^*_p_*_=_ 0.363). LnRMSSD_M_ at PostOL was lower than BL LnRMSSD_M_ (*p* = 0.019, *d* = −0.68). PostTP LnRMSSD_M_ was not different from PostOL (*p* = 0.056, *d* = 0.80) and BL (*p* = 0.998, *d* = −0.10). Compared to BL, LnRMSSD_T_ was not different from PostOL (*p* = 0.590, *d* = −0.41) or PostTP (*p* = 1.000, *d* = 0.00). There was no change in LnRMSSD_T_ between PostOL and PostTP (*p* = 0.590, *d* = 0.43). Figure 1 displays individual changes of LnRMSSD_M_ and LnRMSSD_T_ during BL, PostOL, and PostTP.

### 3.3. Intra-Day LnRMSSD Comparisons

**Figure 2:**
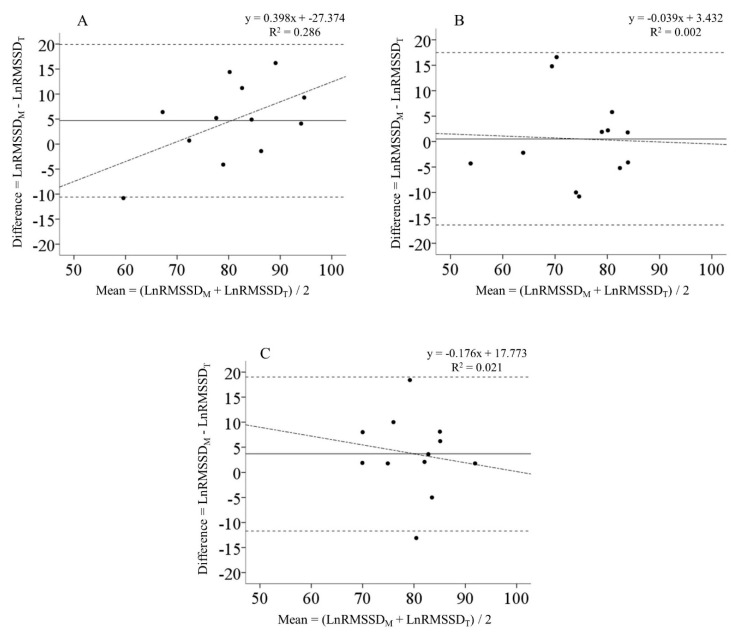
Bland–Altman plots comparing heart rate variability recorded upon waking (LnRMSSD_M_) and heart rate variability recorded upon arriving at the training facility (LnRMSSD_T_) during the (**A**) baseline, (**B**) post-overload, and (**C**) post-taper test sessions. The solid lines represent the mean bias while the horizontal dashed lines represent the 95% limits of agreement. The dashed-dotted regression lines represent the trend between the differences and means.

LnRMSSD_M_ and LnRMSSD_T_ showed large to very large correlations at BL (ICC = 0.71, 95% CI: 0.25–0.91, *p* < 0.001) and PostOL (ICC = 0.65, 95% CI: 0.14–0.89, *p* = 0.010). LnRMSSD_M_ and LnRMSSD_T_ were not related at PostTP (ICC = 0.44, 95% CI: −0.09–0.79, *p* = 0.054). Bland–Altman plots are presented in Figure 2. The constant error (CE) between LnRMSSD_M_ and LnRMSSD_T_ at BL was 4.7 units (lnRMSSDx20) and the 95% limits of agreement varied from −10.6 to 19.9. At PostOL, the CE between LnRMSSD_M_ and LnRMSSD_T_ was 0.5 units and the 95% limits of agreement ranged from −16.4 to 17.5. The CE between LnRMSSD_M_ and LnRMSSD_T_ at PostTP was 3.7 units and the 95% limits of agreement ranged from −11.7 to 19.0. 

### 3.4. Bench Press Performance Comparisons

Bench press 1RM, MCV, and RTF during the introduction, overload, and taper microcycles are displayed in Table 2. There was a significant main effect for bench press 1RM (*p* < 0.001, n^2^*_p_*_=_ 0.672). There was no difference in 1RM between BL and PostOL (*p* = 0.262, *d* = −0.05). Following the taper, 1RM increased above BL (*p* < 0.001, *d* = 0.15) and PostOL (*p* < 0.001, *d* = 0.21). There was a significant main effect for bench press MCV (*p* < 0.001, n^2^*_p_* = 0.478). Following the overload, MCV was lower than BL (*p* = 0.003, *d* = −0.73). MCV at PostTP increased above PostOL (*p* = 0.002, *d* = 0.74), but was not different from BL (*p* = 0.956, *d* = 0.13). There was a significant main effect for bench press RTF (*p* < 0.001, n^2^*_p_* = 0.666). There was no difference in RTF between BL and PostOL (*p* = 0.099, *d* = −0.58). The total repetitions completed at PostTP were higher than BL (*p* < 0.001, *d* = 0.96) and PostOL (*p* < 0.001, *d* = 1.36).

There were no significant associations between ΔLnRMSSD_M_ and Δ1RM, ΔMCV, ΔRTF between BL, PostOL, and PostTP (*p* = 0.087–0.981). Furthermore, ΔLnRMSSD_T_ was not associated with Δ1RM, ΔMCV, ΔRTF across any microcycles (*p* = 0.094–0.871). Correlation coefficients are presented in Table 3.

## 4. Discussion

This study evaluated the changes in smartphone-derived LnRMSSD taken upon awakening in the morning and right before performance testing on three days: (1) before and (2) following a microcycle of overload training and (3) following a taper microcycle in competitive powerlifters. The primary findings were that LnRMSSD_M_ decreased following the overload and returned to baseline following the taper; however, there was no change in LnRMSSD_T_ across each of the test sessions.

The overload microcycle consisted of high-volume and high-intensity resistance training utilizing multi-joint exercises of squat, bench press, and deadlift. Previous investigations have demonstrated that this type of training can cause tremendous homeostatic perturbations that may lead to decrements in performance [28], which may be referred to as overreaching [18]. Raeder et al. found that 6 days of intensified resistance training produced increases in muscle damage indicated by significantly elevated creatine kinase concentrations [28]. Repetitive muscle contractions occurring during high-load, high volume, resistance training can compromise muscle fiber integrity involving damage of the sarcomeres and contractile proteins. Chen et al. noted a significant increase in muscle soreness and creatine kinase following an intense resistance training session in a group of competitive weightlifters [13]. Additionally, the participants’ experienced a significant decrease in HRV (high frequency spectral power) 24 h post-training. 

In the present study, a 4-day overload microcycle was associated with a reduction in LnRMSSD_M_ values compared to BL. While no physiological markers of muscle damage were taken, participants reported significantly lower PRS scores indicating poor recovery and expected decreases in performance. In the investigation by Chen et al., a significant reduction in HRV (high frequency) was mirrored by a non-significant 3 kg decrease in back squat performance 24 h following the training session [13]. Similarly, the present study observed that LnRMSSD_M_ mirrored the changes in bench press performance. The overload microcycle had a large decrease in MCV and a very large decrease in RTF. Flatt and colleagues demonstrated that a high-volume training session produced a very large decrease in perceived recovery and bench press velocity, while supine LnRMSSD experienced a small decrease 24 h following training [29]. While the MCV and RTF were suppressed following the overload, bench press 1RM was unchanged. The discrepancy between changes in bench press velocity and 1RM may be explained by previous observations noting that measures of velocity and power tend to decrease earlier than measures of maximal strength following intensified resistance training. A 3-week high-intensity resistance training program increased 9.6 m sprint times by 6.3% while back squat 1RM increased by 16.0% in resistance-trained males [30]. It is speculated that a longer duration of overload, such as in previous investigations [28], may have produced decrements in maximal strength.

The taper period consisted of reduced training volume and intensity to allow for fatigue dissipation and recovery [17]. During the taper, total volume load and sRPE were significantly lower than overload values. The very large load reduction produced a large increase in LnRMSSD_M_ above overload values. Additionally, a very large increase in participants’ PRS indicated better perceived recovery. Bench press performance mirrored changes in LnRMSSD_M_, as PostTP 1RM, MCV, and RTF were higher than at PostOL. These findings support the results of Chen et al. wherein HRV and weightlifting performance increased following a recovery period [13]. While similar results were observed in the present study, HRV measurements recorded by Chen et al. consisted of 5 min measures utilizing an electrocardiogram. In the present study, HRV was measured via a PWFS and smartphone application which highlights the practicality of the field device. 

Advances in technology allow for more practical athlete monitoring. Smartphone-derived LnRMSSD allows for HRV monitoring to be utilized by coaches and athletes in the field. It has been suggested that HRV measurements be recorded upon waking [4]; however, this approach may not be the most practical. For instance, upon-waking measures require each athlete to have the necessary equipment needed (i.e., heart rate monitor and smartphone/tablet) to record the HRV. An additional concern with upon-waking-measures is the difficulty in daily compliance experienced with most self-recorded measures [31]. Therefore, smartphone-derived LnRMSSD measurements recorded upon arriving at the training facility could be more practical than measures performed upon waking. 

During baseline and overload testing, LnRMSSD_M_ and LnRMSSD_T_ showed very large to large correlations, while no significant association was seen at PostTP. Additionally, Bland–Altman plots showed extremely wide limits of agreement between LnRMSSD_M_ and LnRMSSD_T_, suggesting large individual errors between measurements. Our results conflict with a previous investigation by Nakamura et al. wherein they found a very high reliability of intra-day LnRMSSD measures [32]. However, in their study, intra-day LnRMSSD measures were recorded only 10 min apart and LnRMSSD measures were captured using a portable heart rate monitor and assessed using HRV computer software. In the present study, the time difference between LnRMSSD_M_ and LnRMSSD_T_ for some participants was as large as 10 h. During this time, the participants may have been exposed to non-training related stressors (i.e., academic or work stress) that could have influenced the relationship between LnRMSSD_T_ and LnRMSSD_M_. It appeared that LnRMSSD_M_ was more sensitive to changes in the training loads than LnRMSSD_T_. Therefore, it is recommended that smartphone-derived LnRMSSD should be recorded upon waking, as this method is most sensitive to changes in resistance training loads. 

Previous studies have demonstrated that ultra-short, smartphone-derived LnRMSSD were sensitive to changes in the training loads in competitive athletes [7,8,9]. In collegiate female soccer players, a very large increase (ES = 2.26) in training load resulted in a small decrease (ES = −0.29 to 0.37) in weekly LnRMSSD values [7]. Similarly, a 20% increase in training load during an overload week resulted in a 5.5% decrease in LnRMSSD in collegiate sprint swimmers [9]. However, no performance measures were provided to compare to the changes in HRV. The current study is the first study to examine the effect of smartphone-derived LnRMSSD in powerlifters. The results of this study suggest that smartphone-derived LnRMSSD is sensitive to changes in resistance training loads at the group level; however, individual responses showed high variability. Changes in LnRMSSD were not associated with changes in bench press 1RM, MCV, or RTF across each of the microcycles. Similarly, Flatt et al. found no significant associations between ΔLnRMSSD and Δcounter-movement jump peak power, Δback squat MCV, and Δbench press MCV [29]. Collectively, these findings suggest that individual measures of LnRMSSD may not accurately predict changes in performance. Thus, athletes and coaches should practice caution when using LnRMSSD to autoregulate training.

A potential limitation of this study is that the participants’ previous training methods and recovery status were not determined prior to the introduction microcycle. This may account for some of the intra-individual differences that existed in response to the standardized training program. During the screening process, participants were excluded if they had peaked for a competition within the past 4 weeks, and all participants included in the study were instructed to refrain from any additional exercise. 

The present study examined changes in smartphone-derived LnRMSSD following a 4-day overload and one-week taper. Training strategies for intermediate to advanced strength athletes may include multiple overload weeks before tapering. Thus, future research is warranted to examine weekly changes in smartphone-derived HRV during resistance training programs with longer periods of overload. This approach would allow for calculations of weekly HRV mean and a coefficient of variation. The coefficient of variation of the weekly mean LnRMSSD has been shown to be useful in assessing how individual athletes respond to training [7,9]. Therefore, future investigations should evaluate weekly changes in the LnRMSSD mean and coefficient of variation across a resistance training program consisting of multiple weeks of overload followed by a taper. Furthermore, future research should investigate performance outcomes following a fixed resistance training program compared to an autoregulated program guided by smartphone-derived HRV measures.

## 5. Conclusions

In the current study, smartphone-derived LnRMSSD, recorded upon waking, was sensitive to changes in training load across the overload and taper microcycles. Similarly, bench press performance mirrored the pattern in LnRMSSD_M_. LnRMSSD_T_ was not different across microcycles and the effect sizes were smaller compared to the changes in LnRMSSD_M_. In addition, large individual differences existed between LnRMSSD_M_ and LnRMSSD_T_ at each of the three time points. This suggests that LnRMSSD recordings later in the day should be used with caution if seeking a surrogate to the preferred morning measures. Therefore, smartphone-derived HRV, recorded upon waking, is a non-invasive method of determining preparedness in a group of strength athletes during periods of intensified training. Coaches can use the team data to determine the internal strain imposed on the athletes from training and autoregulate training as warranted. A substantial decline in HRV would provide an early indication of excessive fatigue and a potential decline in performance. Thus, the coach can adjust the team’s training loads to allow for adequate recovery and monitor HRV until it returns to baseline values. While the current study demonstrates that smartphone-derived HRV can provide meaningful data for monitoring team preparedness, the high variability of individual responses in the current study suggests that coaches should be cautious of using smartphone-derived HRV to autoregulate training on an individual basis.

## Figures and Tables

**Table 1:** Resistance training during introduction, overload, and taper microcycles.

	Day 1	Day 2	Day 3	Day 4	Day 5
Introduction microcycle *	Back squat, 3 × 5 Bench press, 3 × 5 RDL, 3 × 8 Standing OHP, 3 × 8 Lat pulldown, 3 × 8	No resistance training	Deadlift, 3 × 3 Bench press, 3 × 5 Leg press, 3 × 8 Seated OHP, 3 × 8 Barbell row, 3 × 8	No resistance training	Baseline testingBack squat, 3 × 3 RDL, 3 × OHP, 3 × 8 Lat pulldown, 3 × 8
Overload microcycle	Back squat, 3 × 9RM Bench press, 3 × 9RM RDL, 3 × 10RM Standing OHP, 3 × 10RM Lat pulldown, 3 × 10RM	Deadlift, 5 × 5RM Bench press, 4 × 7RM Leg press, 3 × 10RM Seated OHP, 3 × 10RM Barbell row, 3 × 10RM	Back squat, 5 × 5RM Bench press, 5 × 5RM RDL, 3 × 8RM Standing OHP, 3 × 8RM Pull-up, 3 × 8RM	Deadlift, 5 × 3RM Bench press, 7 × 3RM Leg press, 3 × 8RM Seated OHP, 3 × 8RM Barbell row, 3 × 8RM	Overload testing
Taper microcycle **	Back squat, 3 × 5 Bench press, 4 × 5 RDL, 3 × 8 Standing OHP, 3 × 8 Lat pulldown, 3 × 8	No resistance training	Deadlift, 3 × 3 Bench press, 3 × 3 Leg press, 3 × 8 Seated OHP, 3 × 8 Barbell row, 3 × 8	No resistance training	Taper testing

RM = repetition maximum; OHP = overhead press; RDL = Romanian deadlift; * All sets performed to rating of perceived exertion (RPE) 6–8; ** Load calculated as 90% and 85% of average load used during days 3 and 4, respectively, of overload microcycle.

**Table 2:** The comparison of the external load, internal load, heart rate variability and bench press performance across microcycles (n = 12).

	Baseline	Overload	Taper
*External Load*			
Total volume–load (kg)	19,039.4 ± 5283.5	52,836.0 ± 12,970.8 ^†^	16,077.2 ± 3806.6 ^§^
*Internal Load*			
Session RPE	5.3 ± 1.2	8.4 ± 0.6 ^†^	3.6 ± 1.5 ^†,§^
PRS	7.9 ± 0.8	5.1 ± 2.1 ^†^	9.0 ± 1.0 ^§^
*Heart Rate Variability*			
LnRMSSD_M_	82.9 ± 13.0	75.0 ± 9.9 ^†^	81.9 ± 7.1
LnRMSSD_T_	78.3 ± 9.0	74.4 ± 10.2	78.3 ± 8.0
*Bench Press Performance*			
MCV (m∙s^−1^)	0.64 ± 0.06	0.58 ± 0.10 ^†^	0.65 ± 0.09 ^§^
1RM (kg)	133.1 ± 40.3	131.0 ± 38.5	139.3 ± 41.1 ^†,§^
Repetitions to failure	11.8 ± 1.5	10.8 ± 1.9	13.6 ± 2.2 ^†,§^

Data presented as the mean ± standard deviation. RPE = rating of perceived exertion; PRS = perceived recovery status; LnRMSSD_M_ = logarithm of the root mean square of successive R–R interval differences recorded in the morning; LnRMSSD_T_ = logarithm of the root mean square of successive R–R interval differences recorded before testing; MCV = mean concentric velocity of load–velocity profile; 1RM = one-repetition maximum. ^†^ Significantly different than baseline (*p* < 0.05). ^§^ Significantly different from PostOL (*p* < 0.05).

**Table 3:** Correlation coefficients for changes (Δ) in LnRMSSD and bench press performance across microcycles.

Performance Metric	ΔLnRMSSD Morning (%)			ΔLnRMSSD Testing (%)		
	BL to PostOL	PostOL to PostTP	BL to PostTP	BL to PostOL	PostOL to PostTP	BL to PostTP
ΔBench press 1RM (%)	r = 0.32	r = 0.11	r = −0.36	r = 0.20	r = 0.33	r = 0.05
ΔBench press MCV (%)	r = −0.06	r = −0.01	r = 0.02	r = 0.50	r = 0.26	r = 0.33
ΔBench press RTF (%)	r = 0.52	r = −0.08	r = 0.06	r = 0.10	r = 0.10	r = 0.08

LnRMSSD = natural logarithm of the root mean square of successive R–R interval differences; BL = baseline; PostOL = post-overload; PostTP = post-taper; 1RM = one-repetition maximum, MCV = mean concentric velocity; RTF = repetitions to failure.

## Data Availability

No new data were created or analyzed in this study. Data sharing is not applicable to this article.

## References

[1] Halson S.L. (2014). Monitoring training load to understand fatigue in athletes. Sports Med..

[2] Scott B.R., Duthie G.M., Thornton H.R., Dascombe B.J. (2016). Training monitoring for resistance exercise: Theory and applications. Sports Med..

[3] Achten J., Jeukendrup A.E. (2003). Heart rate monitoring. Sports Med..

[4] Buchheit M. (2014). Monitoring training status with HR measures: Do all roads lead to Rome. Front. Physiol..

[5] Esco M., Flatt A., Nakamura F. (2016). Initial weekly HRV response is related to the prospective change in VO2max in female soccer players. Int. J. Sports Med..

[6] Tian Y., He Z.-H., Zhao J.-x., Tao D.-L., Xu K.-Y., Earnest C.P., Mc Naughton L.R. (2013). Heart rate variability threshold values for early-warning nonfunctional overreaching in elite female wrestlers. J. Strength Cond. Res..

[7] Flatt A.A., Esco M.R. (2015). Smartphone-derived heart-rate variability and training load in a female soccer team. Int. J. Sports Physiol. Perform..

[8] Flatt A.A., Esco M.R., Nakamura F.Y. (2016). Individual heart rate variability responses to preseason training in high level female soccer players. J. Strength Cond. Res..

[9] Flatt A.A., Hornikel B., Esco M.R. (2016). Heart rate variability and psychometric responses to overload and tapering in collegiate sprint-swimmers. J. Sci. Med. Sport.

[10] Kiviniemi A.M., Hautala A.J., Kinnunen H., Nissila J., Virtanen P., Karjalainen J., Tulppo M.P. (2010). Daily exercise prescription on the basis of HR variability among men and women. Med. Sci. Sports Exerc..

[11] Esco M.R., Flatt A.A. (2014). Ultra-short-term heart rate variability indexes at rest and post-exercise in athletes: Evaluating the agreement with accepted recommendations. J. Sports Sci. Med..

[12] Flatt A.A., Esco M.R. (2013). Validity of the ithleteTM smart phone application for determining ultra-short-term heart rate variability. J. Hum. Kinet..

[13] Chen J.-L., Yeh D.-P., Lee J.-P., Chen C.-Y., Huang C.-Y., Lee S.-D., Chen C.-C., Kuo T.B., Kao C.-L., Kuo C.-H. (2011). Parasympathetic nervous activity mirrors recovery status in weightlifting performance after training. J. Strength Cond. Res..

[14] American College of Sports Medicine (2017). ACSM’s Guidelines fo Exercise Testing and Prescription.

[15] The United States Powerlifting Association. http://www.uspa.net/.

[16] Zourdos M.C., Klemp A., Dolan C., Quiles J.M., Schau K.A., Jo E., Helms E., Esgro B., Duncan S., Merino S.G. (2016). Novel resistance training-specific rating of perceived exertion scale measuring repetitions in reserve. J. Strength Cond. Res..

[17] Murach K.A., Bagley J.R. (2015). Less is more: The physiological basis for tapering in endurance, strength, and power athletes. Sports.

[18] Halson S.L., Jeukendrup A.E. (2004). Does overtraining exist?. Sports Med..

[19] Heathers J.A. (2013). Smartphone-enabled pulse rate variability: An alternative methodology for the collection of heart rate variability in psychophysiological research. Int. J. Psychophysiol..

[20] Wegerif S.C. (2014). Method, System and Software Product for the Measurement of Heart Rate Variability.

[21] Saboul D., Pialoux V., Hautier C. (2013). The impact of breathing on HRV measurements: Implications for the longitudinal follow-up of athletes. Eur. J. Sport Sci..

[22] Laurent C.M., Green J.M., Bishop P.A., Sjökvist J., Schumacker R.E., Richardson M.T., Curtner-Smith M. (2011). A practical approach to monitoring recovery: Development of a perceived recovery status scale. J. Strength Cond. Res..

[23] Hori N., Andrews W. (2009). Reliability of velocity, force and power obtained from the Gymaware optical encoder during countermovement jump with and without external loads. J. Aust. Strength Cond..

[24] Helms E.R., Storey A., Cross M.R., Brown S.R., Lenetsky S., Ramsay H., Dillen C., Zourdos M.C. (2016). RPE and velocity relationships for the back squat, bench press, and deadlift in powerlifters. J. Strength Cond. Res..

[25] Cohen J. (1988). Statistical Power Analysis for the Behavioral Sciences.

[26] Hopkins W., Marshall S., Batterham A., Hanin J. (2009). Progressive statistics for studies in sports medicine and exercise science. Med. Sci. Sports Exerc..

[27] Bland J.M., Altman D. (1986). Statistical methods for assessing agreement between two methods of clinical measurement. Lancet.

[28] Raeder C., Wiewelhove T., Simola R.Á.D.P., Kellmann M., Meyer T., Pfeiffer M., Ferrauti A. (2016). Assessment of fatigue and recovery in male and female athletes after 6 days of intensified strength training. J. Strength Cond. Res..

[29] Flatt A.A., Globensky L., Bass E., Sapp B.L., Riemann B.L. (2019). Heart Rate Variability, Neuromuscular and Perceptual Recovery Following Resistance Training. Sports.

[30] Fry A.C., Webber J.M., Weiss L.W., Fry M.D., Li Y. (2000). Impaired performances with excessive high-intensity free-weight training. J. Strength Cond. Res..

[31] Plews D.J., Laursen P.B., Meur Y.L., Hausswirth C., Kilding A.E., Buchheit M. (2014). Monitoring training with heart-rate variability: How much compliance is needed for valid assessment?. Int. J. Sports Physiol. Perform..

[32] Nakamura F.Y., Pereira L.A., Esco M.R., Flatt A.A., Moraes J.E., Abad C.C.C., Loturco I. (2016). Intraday and interday reliability of ultra-short-term heart rate variability in rugby union players. J. Strength Cond. Res..

